# A Case of Pneumococcal Peritonitis after Caesarean Section in a Healthy Woman

**DOI:** 10.1155/2015/350573

**Published:** 2015-10-13

**Authors:** Georgios Kourounis, Yiannis Panayiotou, Patrick Paul Tabet, Brian David Wensley Richards, Athanasios Petrou, Marios Loizou

**Affiliations:** ^1^St George's University of London Programme, University of Nicosia, 93 Agiou Nikolaou Street, Engomi, 2408 Nicosia, Cyprus; ^2^St George's University of London Medical School, Cranmer Terrace, London SW17 0RE, UK; ^3^Surgery Department, New Nicosia General Hospital, Limassol Old Road No. 215, Strovolos, 2029 Nicosia, Cyprus

## Abstract

Pneumococcal peritonitis is prevalent in children and adults with comorbidities but extremely rare in healthy adults. Here we describe a case of pneumococcal peritonitis in a previously healthy woman with no known risk factors who presented with constipation, abdominal pain, and distention. Her only past medical history was an uncomplicated C-section two months prior to presentation. A laparotomy revealed a pneumococcal peritonitis without visible source of infection. The patient remained hospitalized until completion of antibiotic regimen with Ceftriaxone and resolution of symptoms. This report adds to the small body of evidence showing possible pneumococcal peritonitis in healthy young adults.

## 1. Introduction

*Streptococcus pneumoniae* is a well-known and studied pathogen. It is a leading cause of pneumonia and meningitis worldwide [1, 2] and can rarely be found in the abdominal cavity. Pneumococcal peritonitis is well identified in children [3–7] and in adults with comorbidities [8]. Primary pneumococcal peritonitis in adults is seen in patients with liver cirrhosis [9–12], kidney disease [13], and postgastrointestinal surgery [14], women with genitourinary tract infections [14, 15], and elderly patients with ulcerating gastric diseases [14]. Through our literature review, we were able to find only a few case reports describing primary pneumococcal peritonitis in healthy adults in the absence of any of the previously mentioned conditions [16–18]. Secondary pneumococcal peritonitis is more prevalent and defined as having a clearly identifiable source of infection [16]. It is often linked with prior gastrointestinal infection penetration of the peritoneum [3, 14, 15]. We describe here a healthy woman with pneumococcal peritonitis without a primary source of infection or any comorbidities.

## 2. Case Report

A 31-year-old woman presented at the emergency department with abdominal pain and constipation. The abdominal pain commenced three days prior to admission, the first two of which were accompanied with diarrhea. The patient did not report any prior gynecological, respiratory, or other complaints. Relevant past medical history included an uncomplicated caesarean section two months prior to presentation. She denied any previous illness including genitourinary infections and gastrointestinal disease.

On examination, the patient was alert and oriented. She had a visibly distended abdomen which was extremely tender to palpation. No ecchymosis or hematomas were seen on inspection. The caesarean section site of incision was healed and clean. On admission, her temperature was 36.6°C. She had a heart rate of 133 beats per minute and a respiratory rate of 30 breaths per minute. Her peripheral white blood cell count was 1.18 × 10^3^/*μ*L (1.18 × 10^9^/L) with a high neutrophil count of 85.6%. These findings were indicative of systemic inflammatory response syndrome. The C-reactive protein measure was 53.8 mg/dL (538 mg/L). Bowel sounds were markedly reduced and her rectal examination revealed soft stools with no visible signs of blood in them.

**Figure 1:**
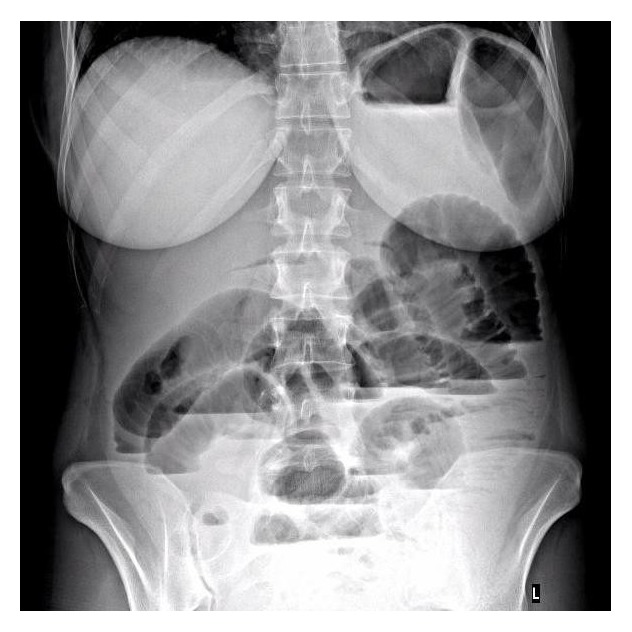
Erect abdominal X-ray on admission with distended bowels showing fluid levels.

After initial investigation with abdominal X-ray showing distended bowel loops with fluid levels (Figure 1), explorative laparotomy was done and revealed pyoperitonitis with patchy inflammation in the pelvis. Ovaries and fallopian tubes appeared edematous and inflamed. Multiple peritoneal lavages were performed and cultures of peritoneal pseudomembranes revealed only* Streptococcus pneumoniae*, three days later. No other microorganisms were found in the peritoneal cultures. Vaginal swab was negative for* Streptococcus pneumoniae*. The GI tract revealed no foci of trauma, necrosis, or perforations. Loops of small bowel appeared intact but edematous. The decision was taken to leave the surgical wound open with a medical vacuum system. The patient was then started on Ceftriaxone, 2000 mg twice a day for 10 days, and was transferred to the intensive care unit (ICU). Surgery was resumed 36 hours later and revealed a distinct reduction in pelvic inflammation in the absence of pus. Only few pseudomembranes remained on the walls of the small intestine and the reduction in abdominal contents edema made closure of the surgical wound possible.

During her stay in ICU, the patient developed fever and diarrhea. Blood cultures and vaginal swabs were negative, as were the stool samples tested for* Clostridium difficile* toxins A and B. In the ICU, the patient also developed bilateral pleural effusions which were investigated by microbiology with negative culture results. Her condition improved with continuation of prior antibiotic therapy. She remained in ICU for a total of 6 days before being transferred to the general surgery ward. The patient was eventually discharged 23 days after admission.

## 3. Discussion

*Streptococcus pneumoniae* is mainly found in the upper respiratory tract of healthy adults and children [1]. The antibiotic era and the acidic environment of the healthy vagina lead to the rarity of* Streptococcus pneumoniae* in the normal vaginal flora [19], making the risk of genital infection with this organism extremely rare [19]. During puerperium and pregnancy, the changes to the pH levels in the vagina encourage a change in normal flora allowing* Streptococcus pneumoniae* to colonize the vagina [19].

Though the route and pathogenesis remain undetermined, it is believed that pneumococci can access the peritoneum via the bloodstream after a pneumococcal respiratory infection, via the lymphatic system and the female genital tract, or transmurally via the gastrointestinal tract [16, 20]. Pneumococcal peritonitis was first described in 1885 by Da Bozzolo [20]. It is classified as either primary or secondary peritonitis and is a surgical emergency. In primary pneumococcal peritonitis there is no other evident site of infection; it is commonly associated with liver and kidney disease [9–13]. Secondary pneumococcal peritonitis is usually caused by a primary gastrointestinal or genitourinary tract infection that penetrated the abdominal cavity [3, 14, 15]. Though rarely seen in adults, primary pneumococcal peritonitis occurs predominantly in women [16]. In our case, the patient had a history of a caesarean section two months prior to presentation; as the postpartum period can last up to 6 months [21], it could have played a role in the pathological process. Another case was reported in a postpartum woman, although in the previous report the women had three previous cesarean sections as sole history that were four, five, and seven years prior to the peritonitis [18].

Bacteremia is a complication frequently seen following a peritoneal infection [16]. This phenomenon was not observed in our patient.

The management of pneumococcal peritonitis includes antimicrobial treatment in combination with surgical intervention. Diagnostic and therapeutic laparotomy should be done to confirm the diagnosis and peritoneal toilet helps to reduce the development of abscess and inflammation [16, 22]. Depending on the cause, both Gram stain and culture may show single or mixed organisms [14, 16]. Analysis of the peritoneal fluid helps in differentiating between primary and secondary peritonitis [16]. Identification of the offending organism and determination of definitive antimicrobial treatment are important due to the increasing prevalence of antibiotic resistant strains of* Streptococcus pneumoniae*. In our case, similar to previous case reports, Ceftriaxone was found efficacious and it should be used, especially in antibiotic resistant strains [16, 23, 24].

In conclusion, pneumococcal peritonitis in adults, although rare, can be observed in previously healthy young adults without comorbidities or visible primary site of infection [16]. It is more frequently seen in females [16]. The nonspecific clinical symptoms make establishment of the diagnosis difficult. Prognosis is good if management involves the combination of adequate antibiotic cover and surgical treatment [16].
